# Copper Micro-Labyrinth with Graphene Skin: New Transparent Flexible Electrodes with Ultimate Low Sheet Resistivity and Superior Stability

**DOI:** 10.3390/nano6090161

**Published:** 2016-09-01

**Authors:** Hak Ki Yu

**Affiliations:** Department of Materials Science and Engineering and Department of Energy Systems Research, Ajou University, Suwon 16499, Korea; hakkiyu@ajou.ac.kr; Tel.: +82-31-219-1680

**Keywords:** Cu micro-labyrinth, transparent flexible electrode, graphene

## Abstract

We have developed self-assembled copper (Cu) micro-labyrinth (ML) with graphene skin for transparent flexible electrodes of optoelectronic devices. The Cu ML is simply formed by heating a thin Cu film with a 100-nm thickness on a SiO_2_/Si substrate at 950 °C under hydrogen ambient to block the oxidation. Moreover, the Cu ML can have graphene skin at the surface by inserting carbo-hydroxyl molecules (C_x_H_y_) during heating due to the catalytic decomposition of C–H bonds on the Cu surface. The Cu ML with graphene skin (Cu ML-G) has superior sheet resistivity below 5 Ω/sq and mechanical flexibility without cracks at the bending radius of 0.1 cm. Although the transmittance of Cu ML-G is a little lower (70%~80%) than that of conventional metallic nanowires electrodes (such as Ag, ~90% at the visible wavelength), it has good thermal stability in conductivity without any damage at 200 °C due to a micro-sized pattern and graphene skin which prohibits the surface migration of Cu atoms.

## 1. Introduction

Recently, due to the development of organic materials and nanotechnology, the research for flexible functionality of optoelectronic devices are attracting great interests [1,2,3]. Among several issues for the development of ultimate flexible optoelectronic devices, the transparent flexible electrodes (TFE) which determine the efficiency of the devices is one of the most important research topics [4,5,6,7,8,9,10]. Although many materials for the TFE have been studied, such as metallic nanowire connection (Au, Ag, and Cu) [4,5], carbon based materials such as carbon nanotube, graphene, and conducting polymers [6,7,8], and conducting oxide nanostructures (Sn doped In_2_O_3_: Indium-Tix-Oxide (ITO) and doped ZnO) [9,10], each material has demerits for the TFE applications.

The metallic nanowire meshes are not suitable for harsh environmental conditions (high temperature and humidity). Because the melting point of nano-sized materials is lower than that of bulk status [11,12], high temperature operation even at 100 °C causes severe migration and disconnection of metallic nanowires, resulting in low conductivity. The carbon-based nano-structures also have an inherent limitation in conductivity (carbon nanotubes: 100~1000 Ω/sq, graphene: 100~10,000 Ω/sq, and PEDOT-PSS: 100~1000 Ω/sq) compared with metallic nanowire (10~100 Ω/sq). Moreover, the conducting oxide nanostructures also have demerits in the limited conductivity itself and weaknesses in acid processes (decomposed easily even with small concentration of H^+^) [13,14]. Meanwhile, we have focused on the composite materials using the above TFE materials. The greatest weakness of the metal nanowire mesh is low thermal stability, but it has the most superior conductivity. On the other hand, the carbon-based materials have good thermal stability, but they have low conductivity, relatively. To increase the thermal stability of metal nanowire, the atomic carbon layer called graphene can be used by wrapping the metal surfaces.

**Figure 1:**
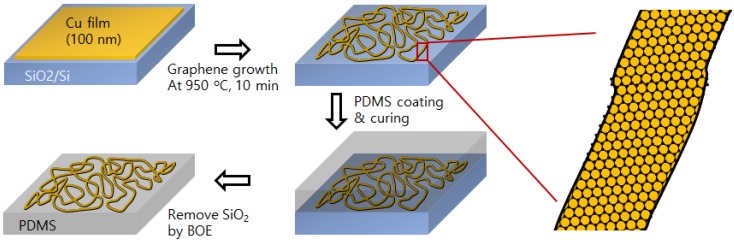
Schematics of the experimental idea: Heating of Cu film (100 nm, on SiO_2_ covered Si) makes a micro-labyrinth pattern. The pattern can have graphene skin at the surface by inserting carbo-hydroxyl molecules (CH_4_ in this experiment). By using polydimethylsiloxane (PDMS) support and SiO_2_ etching technology, the Cu labyrinth pattern with graphene skin can be transferred and used as flexible electrodes.

As shown in Figure 1, thin Cu film (about 100 nm on amorphous substrate) can be easily agglomerated and partially evaporated at a temperature higher than 900 °C at the reduction ambient, resulting in a micro-labyrinth (ML) pattern. The Cu ML can have graphene skin at the surface by inserting carbo-hydroxyl molecules (CH_4_ in this experiment) during heating due to the catalytic decomposition of C–H bonds on the Cu surface. The micro-sized diameter of the Cu labyrinth can have a higher melting point than nano-sized connection, although the transmittance can be degraded. Moreover, the graphene skin can block the surface migration of Cu atoms at the high temperature annealing, resulting in good thermal stability [15]. The graphene skin also support superior mechanical strength when the Cu ML used as extreme bending circumstances (for the flexibility, the Cu ML with graphene skin (Cu ML-G) can be transferred via polydimethylsiloxane (PDMS) support and the SiO_2_ etching process shown in Figure 1).

## 2. Results and Discussion

**Figure 2:**
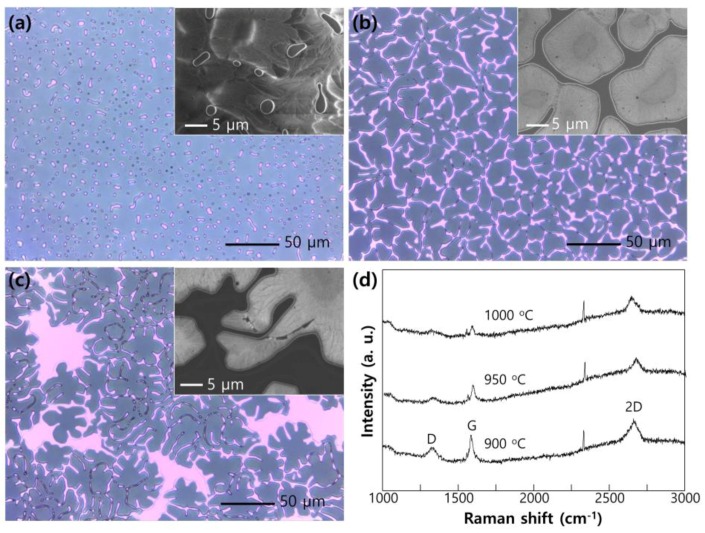
Optical microscope images (each inset is magnified scanning electron microscope (SEM) images) of Cu film on SiO_2_/Si after heating for 10 min with H_2_ (20 sccm) and CH_4_ (6 sccm) ambient (total vapor pressure 460 mTorr) at (**a**) 1000 °C; (**b**) 950 °C; and (**c**) 900 °C; (**d**) Raman spectra of graphene covered copper micro-labyrinth (Cu ML) with respect to heating temperature.

Figure 2 shows optical microscope images (each inset is magnified scanning electron microscope (SEM) images) of Cu film on SiO_2_/Si after heating for 10 min with H_2_ (20 sccm) and CH_4_ (6 sccm) ambient (total vapor pressure 460 mTorr) at (a) 1000 °C, (b) 950 °C and (c) 900 °C. Because the diffusion width of Cu atoms during heating depends more strongly on the diffusivity determined by temperature (Arrhenius relation) than heating time [16], the severe change in morphology was shown among those temperatures. The ideal labyrinth pattern, which should be connected but has the least coverage on the substrate, was acquired by heating at 950 °C. The contents of Cu can be modulated by using different thicknesses of Cu film. If we use thin Cu film (below 100 nm), much of the Cu area is opened and the Cu pattern is disconnected after heating. On the other hand, if we use thick Cu film (more than 100 nm), it will take more time to have an optimized labyrinth pattern during annealing. The existence of graphene on the Cu surface was shown by measuring Raman spectra (Figure 2d). The graphene grown on the Cu area (heated at 1000 °C) shows the properties of monolayer graphene, namely large 2D/G line ratio with low D lines [17]. The spectrum of graphene shows a relatively small 2D/G ratio with increased D lines by reducing heating temperature to 900 °C. The strong D line is related to broken hexagonal symmetry of sp^2^ bonded carbon sheets by forming an island growth of graphene due to low solubility of carbon at reduced temperature [18]. However, for the sample heated at 950 °C, the D line and 2D/G ratio is weak, and it can reasonably be concluded that the monolayer of graphene is covered on Cu ML.

**Figure 3:**
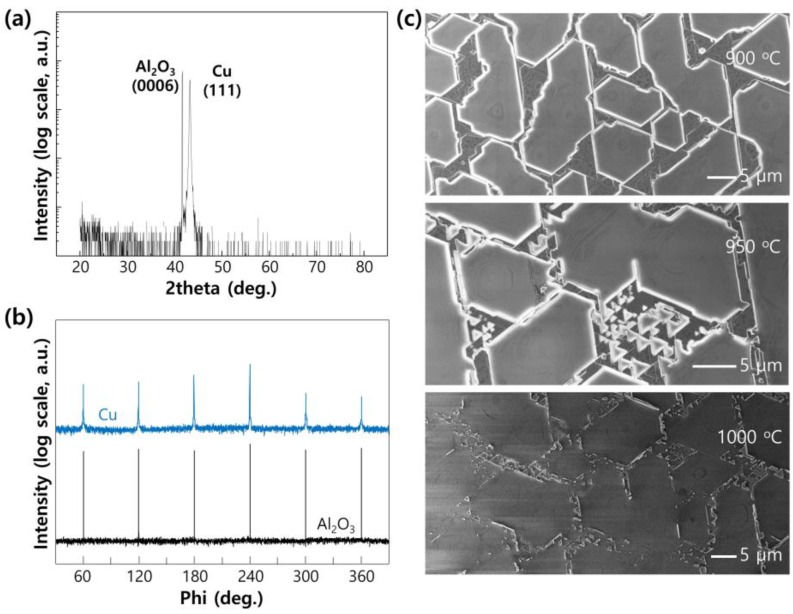
X-ray diffraction of the Cu(111) film (100-nm thickness) on the C-plane sapphire; (**a**) theta-2theta scan with log scale and (**b**) azimuthal scan with fixed θ along the Cu $\left\{2\bar{2}0\right\}$ and sapphire {$3\bar{3}00$} direction. Both exhibit hexagonal symmetry peaks spaced by 60°, which has an epitaxial relationship: $\left(111\right){\left[1\bar{1}0\right]}_{\text{Cu}}∥\left(0001\right){\left[1\bar{1}00\right]}_{C-\text{sapphire}}$; (**c**) SEM images of Cu film on C-sapphire after heating for 10 min with H_2_ (20 sccm) and CH_4_ (6 sccm) ambient (total vapor pressure 460 mTorr) at a temperature of 900~1000 °C.

The Cu ML pattern depends on substrate type that has a different crystal structure and surface-free energy. For the comparison, we have done the same experiments on the C-plane sapphire (0006) substrate. The Cu film (100 nm) grown on C-plane sapphire shows (111) preferred epitaxial orientation—shown in Figure 3a. The atomic distance between Cu (111) surface atoms is 0.255 nm, while the O–O atomic distance in C-plane sapphire is 0.279 nm. The atomic lattice mismatch with 8.6% allows for pseudo-epitaxial growth of Cu (111) plane on C-plane sapphire. The 6-fold symmetry of the Cu (111) plane shown in Figure 3b can be understood as a twin formation of 3-fold Face Centered Cubic (FCC) Cu due to pseudo-epitaxial growth. [19,20]. Figure 3c shows SEM images of the Cu film after heating for 10 min with H_2_ (20 sccm) and CH_4_ (6 sccm) ambient (total vapor pressure 460 mTorr) at 900 °C, 950 °C, and 1000 °C. The tendency for the disconnection of the Cu film and island formation by increasing the heating temperature is similar, but the residual pattern of Cu is totally different. The basic form of retained Cu film is a triangular-fractal pattern, resulting in hexagonal pattern which is related to the 6-fold symmetry measured in Figure 3b. In this work, we have used SiO_2_/Si substrate for the easy transfer of the Cu pattern by etching a SiO_2_ layer.

**Figure 4:**
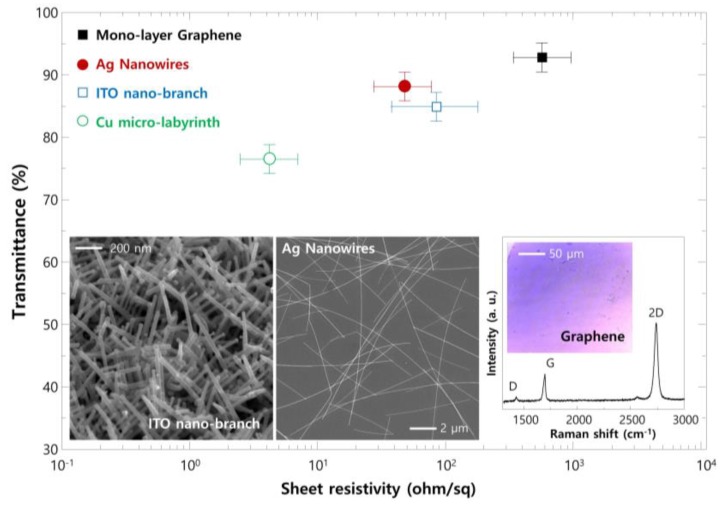
The sheet resistivity and transmittance of Cu ML with graphene skin (Cu ML-G) for the application as transparent electrodes with alternative TFE materials such as mono-layer graphene, Ag nanowires, and indium-tin-oxide (ITO) nano-branches (insets show the SEM images of ITO nano-branches and Ag nanowires; for the monolayer graphene, the Raman spectrum with optical microscope image after transferring to a SiO_2_-covered Si substrate).

**Figure 5:**
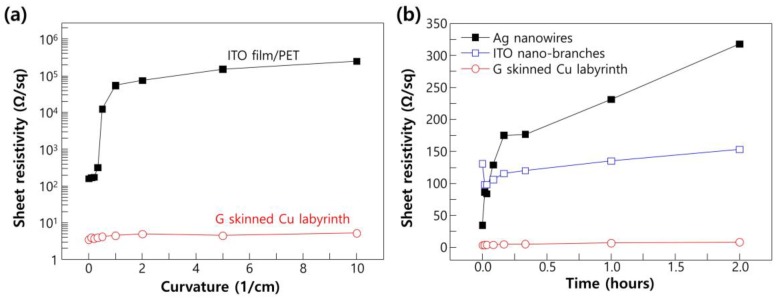
(**a**) Sheet resistivity change of ITO film/polyethylene terephthalate (PET) and Cu ML-G with respect to the various curvatures; (**b**) Change of sheet resistivity with respect to annealing time for the Cu ML-G and alternative transparent flexible electrodes (TFE) materials such as ITO nano-branches and Ag nanowires at 40% humidity and 200 °C temperature conditions.

The sheet resistivity and transmittance of Cu ML with graphene skin (Cu ML-G) for the application as transparent electrodes are shown in Figure 4. The experimental results were compared with alternative TFE such as Mono-layer graphene, Ag nanowires, and ITO nano-branches (insets show the synthesized alternative materials). Compared with these alternative materials, Cu ML-G shows a lower transmittance (moderate about 75%) due to a micro-sized metal pattern; however, it shows low sheet resistivity, even below 3 Ω/sq. Moreover, it shows superior mechanical and thermal stability, which is important for the application to the harsh operation condition of optoelectronic devices (see Figure 5). The mechanical stability was confirmed by carrying out a bending test (Figure 5a) and compared the results against those of conventional ITO film on polyethylene terephthalate (PET), Sigma Aldrich-product number 639281). The relative sheet resistivity of the ITO film degraded after bending at 0.5 cm^−1^ curvature, whereas the electrodes made with Cu ML-G were stable until bending at 10 cm^−1^. The origin of these phenomena is the brittleness of the oxide materials. In ITO film on PET, a severe mechanical crack lines block the electron movement, resulting in an abrupt increase in sheet resistivity, whereas the metal has a flexibility due to ductility. Moreover, the graphene which covers Cu ML itself has excellent mechanical characteristics, including a high tensile strength (~130 GPa) and elastic modulus (~1 TPa) [21].

The thermal stability of Cu ML-G is also superior compared with Ag nanowires and ITO nano-branches (Figure 5b). The sheet resistivity of the Ag nanowire gradually increased with respect to annealing time at 200 °C and 40% humidity, due to the severe disconnection of the junction by Ag atom migration. The migration and agglomeration of Ag nanowire can be carried out at this low temperature because the nano-sized metals have a lower melting point than its bulk status. The ITO nano-branches have relatively stable resistivity, even at long-term thermal annealing. The initial decrease in the sheet resistivity of ITO nano-branches can be understood as the evaporation of water (H_2_O) molecules on the ITO surface, which were adsorbed at the normal ambient. The adsorbed water molecules on ITO surface tend to capture electrons, making the ITO nano-branches less conductive [22]. When the heat is supplied to ITO surface, water molecules can be evaporated and release the captured electrons, thereby recovering the conductivity of the ITO nano-branches. The sight increase in resistivity of ITO nano-branches after long-term annealing can be understood as a loss of metallic indium, which was located in the nano-branch junction. Compared with Ag nanowires and ITO nano-branches, the Cu ML-G shows stable sheet resistivity. Because the pattern size is micro-range, there is no significant drop in the melting point of the Cu metal. Moreover, the graphene skin on the Cu surface hinders the movement of Cu atoms for agglomeration, resulting in superior thermal stability.

## 3. Materials and Methods

**Cu film preparation:** Thermal SiO_2_ (200 nm) covered Si (100) and C-plane sapphire was used as a starting substrate. After cleaning sequentially with acetone, isopropyl alcohol, and de-ionized water, Cu films were deposited by electron beam deposition using a high purity Cu source from Sigma-Aldrich (item number: 254177, Cu beads, 2–8 mm, 99.9995% trace metals basis). The Cu films were grown to a 100-nm thickness at a rate of 0.03 nm/s. The growth chamber pressure was maintained at about 10^−6^ Torr during deposition, and the substrate was held at room temperature.

**Cu micro-labyrinth with graphene skin:** The Cu film/sapphire substrate was loaded into a quartz tube CVD chamber. (1) The pressure in the quartz chamber was pumped down to 3 mTorr using a mechanical pump; (2) A 40-sccm flow of hydrogen gas was inserted to the chamber at 950 mTorr; (3) The Cu films were heated to the target temperature 900~1000 °C; (4) A 6-sccm flow of methane gas with 20 sccm of hydrogen was introduced into the chamber for 10 min (total pressure: 460 mTorr) for the graphene growth on Cu surface; after graphene growth the furnace was cooled down to the room temperature under a continuous 20-sccm flow of hydrogen to block oxidation of Cu.

**Transfer of Cu ML-G:** (PDMS) elastomer (Sylgard 184, Dow Corning) was prepared by a mixing base and curing agent in a 10:1 weight ratio and curing at 70 °C for >2 h or at room temperature for >12 h. Then, the SiO_2_ layer was removed by buffer oxide etch (BOE) for 5 min.

**Preparation of alternative TFEs:** We had prepared the alternative TFE materials based on the previous experimental results [9].

## 4. Conclusions

In this study, graphene-skinned Cu ML were fabricated for the application of TFE by simple heating of Cu film on SiO_2_/Si at the ambient of H_2_ and CH_4_. The Cu ML-G can be transferred by PDMS support and SiO_2_ etching process. The Cu ML-G has superior sheet resistivity below 5 Ω/sq and mechanical flexibility, preventing any cracks up to a 0.1-cm bending radius. Although the transmittance of Cu ML-G is a little lower (70%~80%) than that of commercial metallic nanowires electrodes, it has superior thermal stability in conductance up to 200 °C due to a micro-sized pattern and graphene skin, which prohibits the surface migration of Cu atoms at high temperature. We believe that the Cu ML-G can be used very soon as suitable electrode systems for flexible optoelectronic devices under severe operation circumstances.

## Abbreviations

The following abbreviations are used in this manuscript:

Cu ML	Copper Micro-Labyrinth
Cu ML-G	Copper Micro-Labyrinth with Graphene skin
ITO	Indium-Tix-Oxide
TFE	Transparent Flexible Electrodes
